# Distinct roles of the Gcn5 histone acetyltransferase revealed during transient stress-induced reprogramming of the genome

**DOI:** 10.1186/1471-2164-14-479

**Published:** 2013-07-16

**Authors:** Yongtao Xue-Franzén, Johan Henriksson, Thomas R Bürglin, Anthony PH Wright

**Affiliations:** 1 Clinical Research Center, Department of Laboratory Medicine, Karolinska Institutet SE-141 86 Huddinge, Sweden; 2 Department of Bioscience and Nutrition, Karolinska Institutet, SE-141 86, Huddinge, Sweden; 3 Center for Biosciences, Karolinska Institute, SE-141 86, Huddinge, Sweden

**Keywords:** Gcn5, Gene length, Transcription elongation, Histone acetyltransferase, Stress, Genome-wide association study

## Abstract

**Background:**

Gcn5 belongs to a family of histone acetyltransferases (HATs) that regulate protein function by acetylation. Gcn5 plays several different roles in gene transcription throughout the genome but their characterisation by classical mutation approaches is hampered by the high degree of apparent functional redundancy between HAT proteins.

**Results:**

Here we utilise the reduced redundancy associated with the transiently high levels of genomic reprogramming during stress adaptation as a complementary approach to understand the functions of redundant protein families like HATs. We show genome-wide evidence for two functionally distinct roles of Gcn5. First, Gcn5 transiently re-localises to the ORFs of long genes during stress adaptation. Taken together with earlier mechanistic studies, our data suggests that Gcn5 plays a genome- wide role in specifically increasing the transcriptional elongation of long genes, thus increasing the production efficiency of complete long transcripts. Second, we suggest that Gcn5 transiently interacts with histones close to the transcription start site of the many genes that it activates during stress adaptation by acetylation of histone H3K18, leading to histone depletion, probably as a result of nucleosome loss as has been described previously.

**Conclusions:**

We show that stress adaptation can be used to elucidate the functions of otherwise redundant proteins, like Gcn5, in gene transcription. Further, we show that normalization of chromatin-associated protein levels in ChIP experiments in relation to the histone levels may provide a useful complement to standard approaches. In the present study analysis of data in this way provides an alternative explanation for previously indicated repressive role of Gcn5 in gene transcription.

## Background

Changes in epigenetic marks, such as histone acetylation, are critical for normal biological function and defects in epigenetic programming are associated with cancer [1,2]. For example, a general reduction of acetylated histone H4K16 (H4K16ac) and tri-methylated histone H4K20 has been reported for many cancer types [1]. Further, reduced levels of histone H3K4 di-methylation and acetylated histone H3K18 (H3K18ac) have been associated with a higher risk of prostate cancer recurrence, as well as poor survival rates in both lung and kidney cancer patients [2]. Histone modification enzymes, such as histone deacetylases (HDACs) and histone acetyltransferases (HATs), have thus been suggested as promising drug targets with potential for therapeutic applications [3]. It is therefore important to understand the specific roles played by different enzymes involved in epigenetic programming.

The Gcn5 protein is one of the best characterised HATs. Gcn5 performs both global and locus-specific histone acetylation, as well as acetylation of non-histone proteins such as transcriptional factors [4]. Gcn5 is the catalytic subunit of several related HAT complexes, notably the SAGA complex [5]. The structure and function of Gcn5 is evolutionarily conserved. The protein is found throughout eukaryotic organisms and the human HAT domain can functionally replace the equivalent domain in the yeast protein [6]. Gcn5 is also involved in a conserved sub-set of stress responses in evolutionarily divergent yeast species [7]. Yeast is thus a useful tool for understanding the basic functions of eukaryotic Gcn5 proteins.

Gcn5 has been reported to play a range of different functions associated with transcription. The protein was originally characterised as a transcriptional co-activator[8], which is thought to be recruited to regulatory gene regions where it contributes to gene activation by acetylating key lysines on histones, notably histones H3 and 2B [5,9]. Enrichment of Gcn5 has also been associated with genes that are repressed during stress [7,10,11], but no mechanism for how Gcn5 contributes to gene repression has been characterised. Genome-wide studies showed that Gcn5 is also present throughout the transcribed regions of genes and there is evidence that the Gcn5 protein is important for transcriptional elongation [12-14]. Further work is needed in order to gain an overall picture of how Gcn5 contributes to gene transcription and in particular to understand which aspects of Gcn5 function contribute to gene regulation.

Functional studies of HATs are hampered by the considerable levels of redundancy that are seen between different HAT proteins under many of the conditions that have been studied [15-17]. Conventional approaches studying the loss of functionality associated with gene mutations do not reveal complete information about redundant functions since the proteins that remain adapt their function to compensate for absent protein(s). Thus only a relatively small number of genes require Gcn5 for their expression even though Gcn5 is widely spread throughout the genome [7,10]. It has previously been shown that the level of inter-HAT redundancy is strongly reduced during stress adaptation in yeast [15]. During stress adaptation in the budding yeast, *Saccharomyces cerevisiae*, there is a redistribution of Gcn5 from short genes to the ORFs of long genes [7]. In this work we use a stress and adaptation growth regime to further investigate this phenomenon as well as other aspects of Gcn5 function in gene transcription.

## Results

### Measurement of changes in the genome-wide localisation of Gcn5 and histone acetylation during stress adaptation and recovery

**Figure 1:**
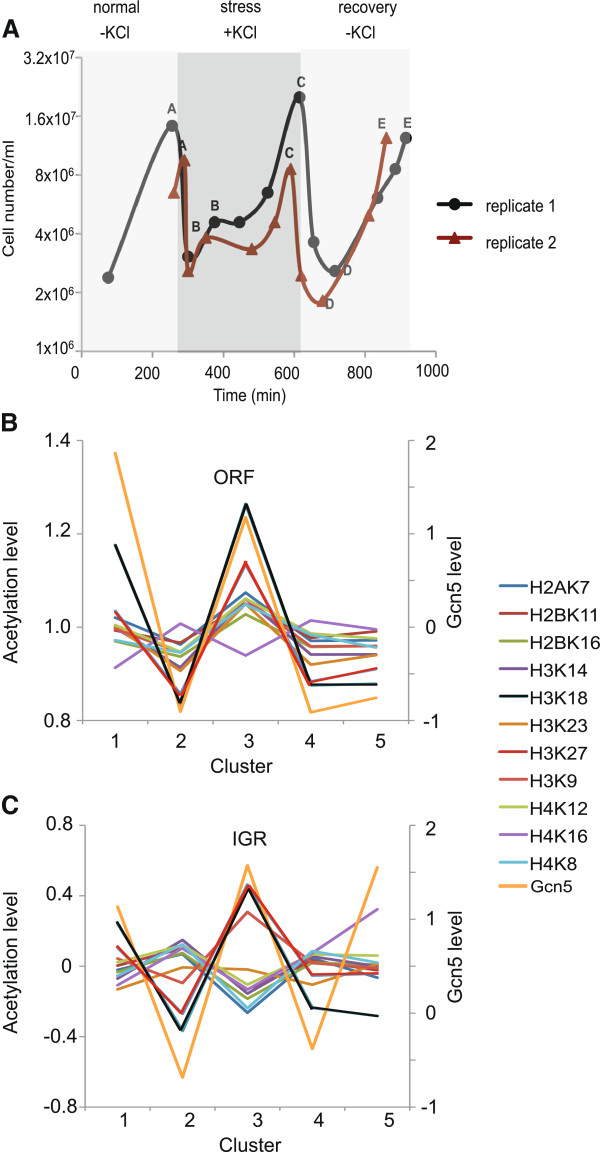
**Growth and sampling of cells during the stress and adaptation growth regime and choice of histone acetylation marks for study. A** Growth curves of cells during the stress and recovery growth regime. Growth curves for two independent experiments show cell growth, the dilution of culture into and out of KCl (1M) containing media and points at which samples **(A**-**E)** were removed for analysis by genome-wide gene expression profiling and ChIP-on-chip analysis. Cells were maintained at a density between 10^6^/ml and 2×10^7^/ml throughout. **B-C** Average levels of Gcn5 and acetylated lysine residues in histones for 5 groups of genes with different characteristic Gcn5 intra-genic distributions (the 5 gene clusters are based on previous publication [7], which are either high at ORF (cluster 1), low at ORF (cluster 2), high at both ORF and IGR (cluster 3), high at 3′IGR (cluster 4), or high at 5′IGR (cluster 5)). Gcn5 and histone acetylation levels for this 5 gene clusters are plotted separately for ORF **(B)** and IGR **(C)** regions.

To study Gcn5 function during the adaptation of cells to new growth states and in relation to gene length, we established a growth regime involving stress adaptation and recovery as shown by the growth curves in Figure 1A. Samples were taken for ChIP-chip analysis and gene expression profiling at different time points during the regime. The first sample (sample A) consisted of exponentially growing cells harvested immediately prior to induction of stress by dilution of cultures into fresh media containing 1M KCl. A second sample was taken 1 hour later during the stress adaptation phase (sample B) after which the cells were allowed to reach a post adaptation phase prior to further sampling (sample C). The cells were then diluted into fresh media without KCl and allowed to recover from the stress treatment. Two more samples were taken one hour after dilution during the recovery process as well as at a later time when recovery was judged to be complete (samples D and E, respectively).

To select histone acetylation marks for the study we used published microarray data from Kurtistani *et al.*[18], which contains acetylation patterns for 11 lysine residues in histones, to find sites that correlate with previously published Gcn5 ChIP-on-chip tiling array data [7]. We have previously shown that Gcn5 localisation patterns on genes can be defined by the patterns found in five main groups of genes. Two classes show Gcn5 enrichment in the 5′ or 3′ inter-genic sequence (IGR) respectively, while a further two classes show high or low Gcn5 levels in the open reading frame (ORF) region of genes. In the fifth class Gcn5 is equally distributed throughout genes. By comparing average acetylation levels for different acetylation sits and Gcn5 enrichment for the ORFs of the 5 gene classes (Figure 1B), we found that most acetylation sites show a similar trend to Gcn5 (yellow), with H3K18ac (black) and H3K27ac (red) being the clearest examples. H4K16ac (purple) shows the opposite trend to Gcn5. Within IGR regions the majority of acetylation sites show the opposite pattern compared to Gcn5. The exceptions are H3K18ac (black), H3K27ac (red) and H3K9ac (orange) (Figure 1C). We chose to study H3K18ac as an example of the modifications that are well correlated with Gcn5 localisation in both the ORFs and IGRs of genes. H4K16ac, which tends to be negatively correlated with Gcn5 localisation in both ORFs and IGRs, was chosen as a control mark that we would expect to be less well correlated with Gcn5 in the study. The overall level of histone H3 (H3) was measured to give an indication of the density of histones and nucleosomes in different chromosomal regions.

### Gcn5 in the ORF region of genes transiently relocalises from short to long genes during stress adaptation

**Figure 2:**
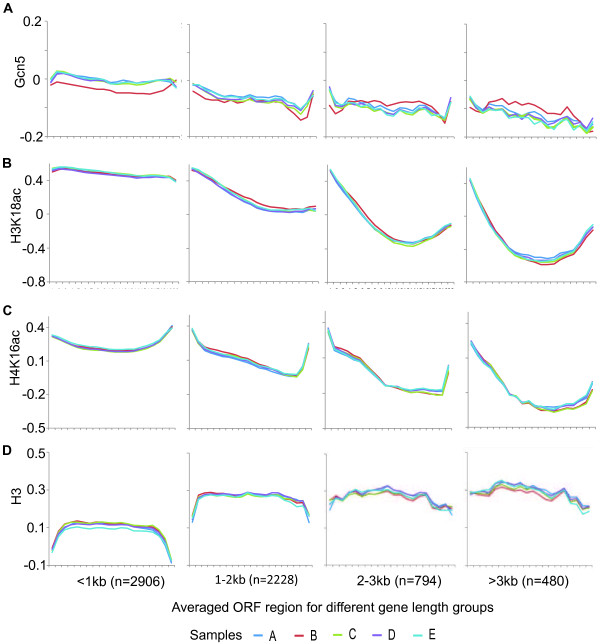
**Genome-wide gene length associated differences in average levels of Gcn5, H3K18ac, H4K16ac and H3 at ORF in samples taken during the stress and recovery growth regime.** The relative average levels (arbitrary units) of Gcn5 **(A)**, H3K18ac **(B)**, H4K16ac **(C)** and H3 **(D)** at ORF are plotted. The arbitrary units are independently defined for each of parts **A**-**D**. The criteria defining the gene-length groups are shown. The number of genes in each group is shown in parenthesis. Line colors represent different samples taken during the stress and recovery growth regime.

We previously reported that Gcn5 in ORFs tends to relocalise from short to long genes under stress conditions. An important question was to determine whether this change is an adaptation associated with long term growth on KCl, or whether it is a transient change associated with the process of adaptation between growth states. To answer this question we analysed the localization of Gcn5 at the five different sample times described above in relation to gene length. Figure 2A shows that Gcn5 levels tend to be lower on the ORFs of long genes compared to short genes. As reported previously [7] the relative localization of Gcn5 tends to shift from short to long gene ORFs during KCl stress adaptation (sample B). Importantly, the shifted pattern is not seen in sample C, where cells have become adapted to growth under KCl stress conditions. The average Gcn5 level that is associated with samples A and C is also seen for samples C and D and we therefore conclude that the transient relocalisation of Gcn5 is specific for the stress adaptation phase and that it is not required for steady-state growth during KCl imposed stress. The transient change in the ORF level of Gcn5 during stress adaptation within different gene-length categories is statistically significant for short and long gene categories (<1 kb, p=2.69E-6; 2-3 kb, p=2.30E-5; >3 kb, p=4.05E-88) but not for the category of intermediate gene length (1-2 kb, p=0.10). Data analysed was taken from the central 40% of each ORF to prevent contamination of signals from flanking inter-genic regions. Redistribution of Gcn5 to the ORFs of long genes during stress adaptation was confirmed by ChIP-qPCR studies of specific long genes (YHR023W, 5786bp; YKL092C, 3315bp; YNL192W, 3396bp; YLR054C, 2262bp), see Additional file 1.

### Reduced Gcn5 occupancy on the ORFs of many short genes during stress adaptation is associated with reduced histone H3K18 acetylation

Next we tested whether transient Gcn5 re-localisation is associated with similar changes in histone acetylation. Figure 2B and C show that the acetylation levels in ORFs of both H3K18 and H4K16 reduce as gene length increases, similar to the pattern observed for Gcn5. This gene length dependent reduction in the average levels of histone acetylation in ORF regions appears to be general for many acetylation sites (Additional file 2). Conversely, H3 levels tend to be higher on longer genes (Figure 2D) and so the length dependent change in histone acetylation per histone is less than for overall acetylation levels even though the trend is still clear. Most importantly, we did not observe a measurable gene length dependent difference in H3K18ac and H4K16ac between cells sampled at different points of the stress and recovery growth regime either without normalisation in relation to H3 levels (Figure 2B and C) or with H3 normalisation (Additional file 3). We conclude that either there is no change in the histone acetylation marks studied that correspond to the changes in Gcn5 or that we have not been able to detect such changes for any of a number of possible reasons.

To increase the chance of observing changes in histone acetylation that correlate with changes in Gcn5 localisation we used non-parametric ANOVA to identify the sets of gene ORFs that were most significantly (p<0.05) changed between conditions in

**Figure 3:**
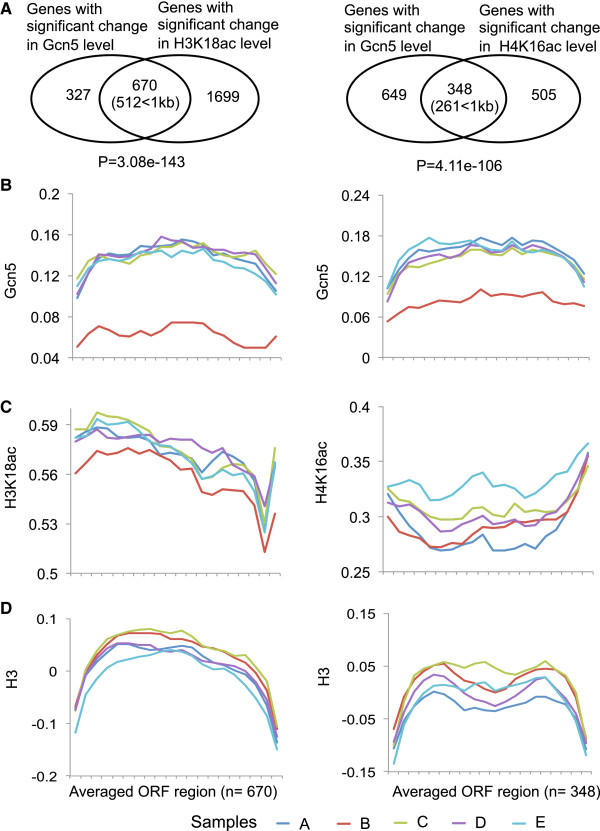
**Transient changes in levels of Gcn5 and H3K18ac during stress adaptation at ORF are correlated for a subset of genes.** A Significant overlap between gene ORFs with significantly (p < 0.05) changed Gcn5 localisation during the stress and recovery growth regime and genes ORFs with significantly (p < 0.05) changed H3K18ac (left panel) or H4K16ac (right panel) levels. The Venn diagrams show the number of overlapping and non-overlapping genes. The p-values show that the number of genes in the intersection is significantly greater than the number expected by chance. The overlapping genes (in parenthesis) are clearly overrepresented in the category of genes shorter than 1kb with p value of 1.73e-99 for the 670 intersection genes and 9.33e-45 for the 348 intersection genes after analysis by a hypogeometric test. **B**-**D** Transient reduction in the average level of Gcn5 during stress adaptation is accompanied by transient reduction in the levels of H3K18ac but not H4K16ac at ORF. The relative average levels (arbitrary units) of Gcn5 **(B)**, H3K18ac **(C)**, H4K16ac **(C)** and H3 **(D)** at ORF are plotted for the groups of overlapping genes defined in part A. The arbitrary units are independently defined for each of parts **A**-**D**. The number of genes in each group is shown in parenthesis. Line colors represent different samples taken during the stress and recovery growth regime.

Gcn5, H3K18ac or H4K16ac over all the conditions sampled. The set of genes with the most significant changes in Gcn5 enrichment (n=997) showed highly significant overlaps with the sets of genes with the most significant changes in H3K18ac (n=2329) and H4K16ac (n=853) (Figure 3A). Further, the overlapping genes show significant overlap with the category of short genes that are less than 1kb in length (Figure 3A). We reasoned that any association between stress adaptation dependent Gcn5 re-localisation in ORFs and histone acetylation would most likely be observable in one or both sets of overlapping genes. Figure 3B shows the average gene Gcn5 levels for the different samples (A-E) for the overlap gene sets identified in Figure 3A. There is a clear transient reduction in Gcn5 levels in ORFs for both gene sets during stress adaptation (sample B) consistent with the transient depletion of Gcn5 on short genes. Figure 3C (left panel) shows that there is a similar tendency for a transient depletion of H3K18ac levels in the ORFs of the overlapping gene set during stress adaptation while there is no clearly detectable change for the H4K16ac gene set (Figure 3C right panel). Figure 3D shows the corresponding levels histone H3 for the selected sets of genes. The lower magnitude of the change in stress adaptation levels of H3K18ac compared to Gcn5 may indicate that there are alternative acetylation mechanisms, such as other HATs, which can compensate for the transient loss of Gcn5 on short genes. The overlapping gene sets contain very few long genes. While both the Gcn5 and H3K18ac re-localisation gene sets show a tendency for transient stress adaptation specific re-localisation to long genes, the genes involved do not overlap and therefore Gcn5 may be involved in the acetylation of a different target on long genes (data not shown).

### Regulated genes under stress adaptation do not show gene-length bias

**Figure 4:**
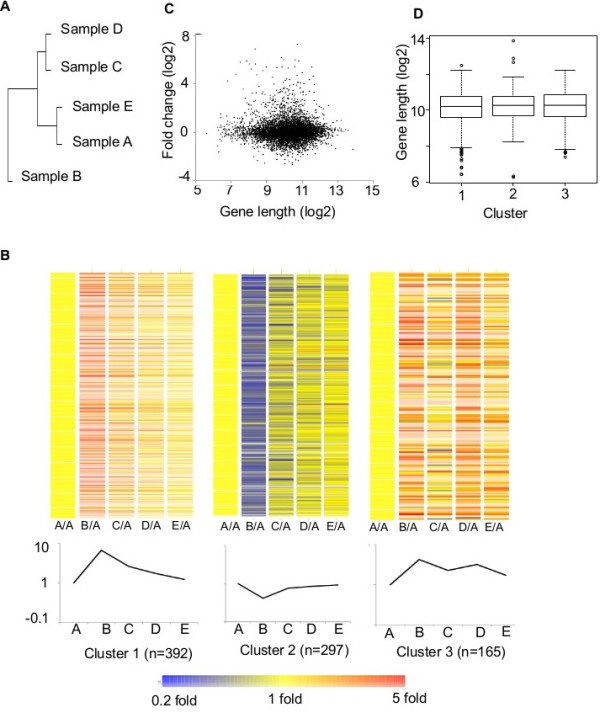
**Gene expression patterns during stress adaptation and recovery are not correlated with gene-length. A** Dendrogram showing the extent of overall RNA abundance level differences in samples collected during the stress and recovery growth regime. The 854 genes that show a change of at least 1.8-fold in at least one of samples **B**, **C**, **D**, and E in relation to sample A were included in the analysis. **B** Genes regulated during the stress and recovery growth regime can be grouped into clusters showing three main patterns of gene expression. Heat maps showing fold change values for genes in samples A-E in relation to their starting level (sample A), for each of the three k-means clusters identified from the 854 regulated genes identified in part A. Log2 transformed fold change values are represented by colors in the heat map and the color range of the heat map is shown by the scale bar. The average expression level values for each sample and cluster are shown in the lower panels and the number of genes in each cluster is shown in parenthesis. **C** Scatter plot showing the lack of any discernable correlation between gene regulation changes (stress adaptation sample B vs normal conditions sample A) and gene length. Log2 transformed fold change values are plotted against Log2 transformed gene length values. Spearman’s rank correlation coefficient rs= 0.0095 (p=0.4739). **D** Box plot shows difference in gene length distributions for the 3 clusters of regulated genes. The distribution of gene length (log2) for the 3 clusters of genes is shown. The median value is shown by black line.

A trivial explanation for the gene length dependent effects we have observed in ORFs would be that gene regulation during stress adaptation is correlated with gene length. To determine whether gene regulation is related to gene length during stress adaptation we performed gene expression profiling on RNA samples collected at the different sampling points in the stress and recovery regime (Samples A-E). A total of 854 genes were up or down regulated more than 1.8 fold in one or more of Samples B-E in relation to their level in Sample A. Overall comparison of these genes shows that the KCl-stress adaptation sample (Sample B) is most different from the other samples while Samples A and E, both representing steady-state growth on normal media, are most similar (Figure 4A).

In order to group the 854 genes into sub-groups characterised by their expression pattern through the stress and recovery regime we performed K-means clustering. Figure 4B shows that the genes cluster into three main groups and that the composition of the clusters is strongly influenced by gene expression patterns during stress adaptation (Sample B). Cluster 1 and Cluster 2 contain genes that are respectively induced or repressed during stress adaptation (Sample B). However, Cluster 3 contains genes that are induced both by stress adaptation (Sample B) and recovery from stress (Sample D). Genes in clusters 1 and 3 are over-represented in over-lapping but distinct sets of gene ontologies categories (Additional file 4). The cluster 1 categories tend to be more focused on stress response and metabolic processes while cluster 3 contains many categories related to stress response and ion transport. Cluster 2 genes, on the other hand, tend to be over-represented in categories related to the protein synthesis capacity of cells. The down-regulation of these genes may thus account for the pause in growth that occurs during stress adaptation.

**Figure 5:**
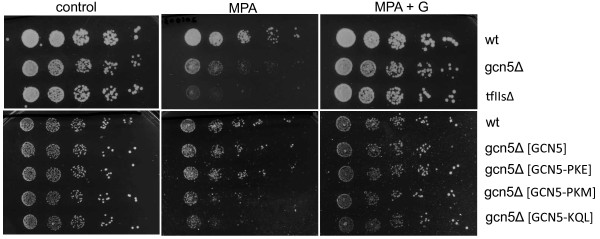
**The histone acetyltransferase activity of Gcn5 is required for transcriptional elongation.** The figure shows serial dilutions of wild type (wt) and mutant (gcn5Δ, tfIIsΔ, and gcn5Δ expressing different gcn5 HAT domain mutants) yeast cells spotted on normal growth media (control) or media containing the transcription elongation inhibitor, mycophenolic acid (MPA), or MPA together with extra Guanine (G), which counteracts the inhibition activity of MPA. Gcn5 derivatives expressed from plasmids are shown in square brakets: [GCN5] – wild type GCN5 and [GCN5-PKE] - HAT domain mutant with full catalytic activity as well as [GCN5-KQL] and [GCN5-PKM] HAT domain mutants with strongly reduced histone acetyltransferase activity (see [9]). TFIIS is an important basal transcription factor involved in transcriptional elongation.

The scatter plot in Figure 4C shows that there is no significant correlation between changes in RNA levels during stress adaptation and gene length when values for all genes are analysed (rs=0.0095, p=0.4739). A similar conclusion can be drawn if only the set of 854 regulated genes is analysed (rs=−0.08, p=0.2). Further, there is no discernable variation in the gene-length distribution for the three regulated gene clusters (Figure 4D). The role of Gcn5 on ORFs is likely to be connected to transcriptional elongation, in which Gcn5 has been suggested to play a role previously [12,14,19]. To confirm this and to determine whether the acetyltransferase activity of Gcn5 is required, we studied the sensitivity of strains lacking Gcn5 or expressing forms of the protein containing mutations in the HAT domain to Mycophenolic Acid (MPA). Mutants with transcriptional elongation defects are generally sensitive to MPA. Figure 5 shows that the strain lacking Gcn5 shows similar MPA sensitivity to a strain lacking the TFIIS transcriptional elongation factor. As expected, the sensitivity is overcome on plates containing guanine that relieves the MPA-mediated depletion of essential nucleotides. Mutants that decrease HAT activity (PKM and KQL) show similar MPA sensitivity to cells lacking Gcn5 while a HAT domain mutant that does not affect HAT activity (PKE) shows higher levels of MPA resistance. We conclude that the HAT activity of Gcn5 is required for transcriptional elongation and that this function may account for the localisation of Gcn5 on ORFs during the stress and recovery growth regime.

### Transiently altered levels of Gcn5 and histone H3K18ac at promoter regions correlate with gene regulation during stress adaptation

**Figure 6:**
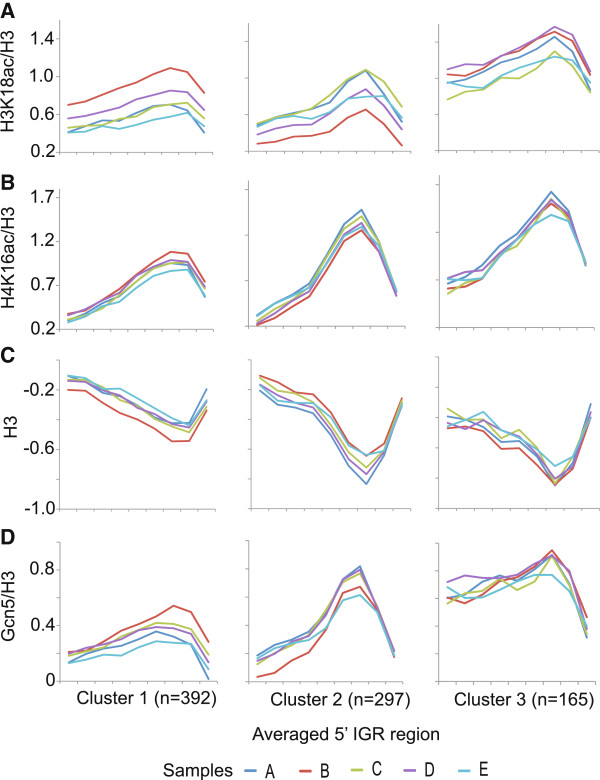
**Transient changes in gene expression during stress adaptation are associated with transient changes in the average levels of Gcn5 and histone acetylation relative to histone levels at promoters.** The relative average levels (arbitrary units) of H3K18ac **(A)**, H4K16ac **(B)**, H3 **(C)** and Gcn5 **(D)** at promoter are plotted. The arbitrary units are independently defined for each of parts A-D. H3K18ac, H4K16ac and Gcn5 levels are shown relative to H3 levels (see Additional file 4 for levels without normalization for H3 levels). Clusters 1–3 are the same as those identified in Figure 4 and the number of genes per cluster is shown in parenthesis. Line colors represent different samples taken during the stress and recovery growth regime.

The lack of any observable correlation between gene regulation and gene length prompted us to analyse Gcn5 and histone acetylation levels at promoter regions, which are expected to be involved in gene regulation. Surprisingly, there was no apparent change in the normalized Gcn5 levels at promoters for the three clusters of genes that are regulated during the adaptation and recovery growth regime (Additional file 5). Contrastingly, the average level of H3K18ac changes in a way that reflects the gene regulation patterns associated with clusters 1–3. After correction for histone density (estimated by measurement of H3 levels) H3K18ac peaks upstream of the ATG at a position consistent with the transcription start site (Figure 6A). This peak is due to the presence of a H3 depleted region since no peak is observed in the uncorrected H3K18ac data. Nucleosome depleted regions in the vicinity of the transcription start site have been described previously [20]. The levels of H4K16ac show a similar pattern to H3K18ac except that the extent of the differences between different samples from the stress and recovery growth regime is lower and the acetylation signals are more clearly concentrated in the promoter peak (Figure 6B). In this case the peak is evident even in the uncorrected data in which there are no observable condition-dependent differences, suggesting that the relatively small changes of H4K16ac in the corrected data are entirely due to changes in histone density (Additional file 5).

**Figure 7:**
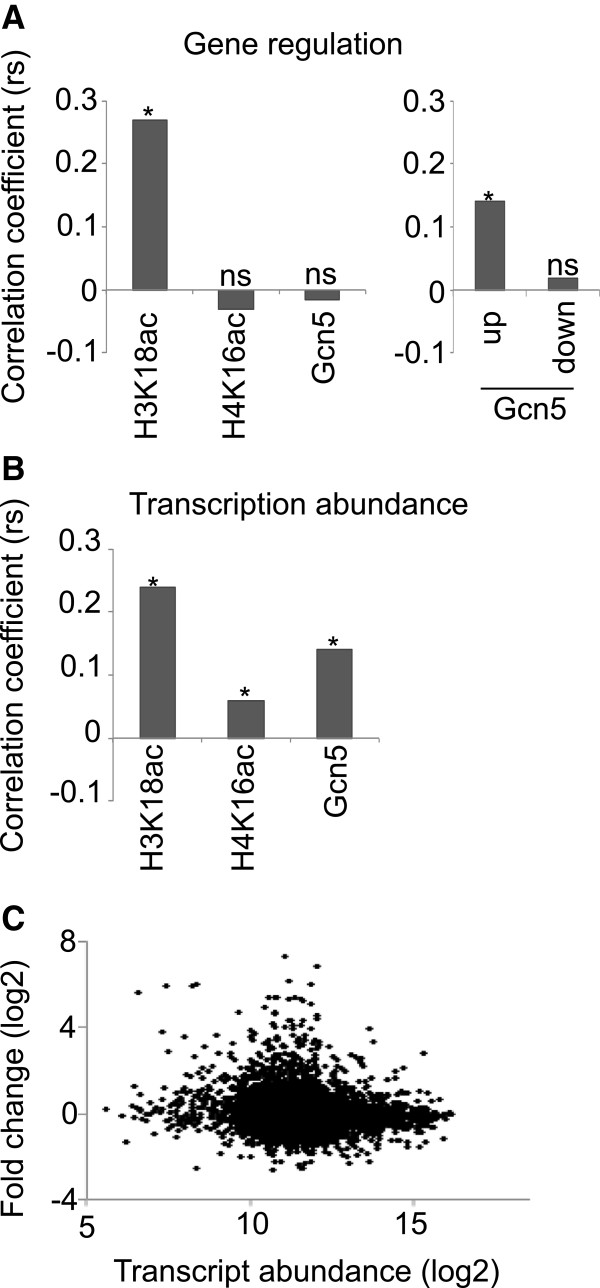
**The level of transient gene activation during stress adaptation is significantly correlated with the extent of the transient increase in H3K18ac and Gcn5 on corresponding promoters. A** Significant correlation between the extent of gene regulation during stress adaptation and levels of increased H3K18ac as well as between gene activation and Gcn5 levels that occur at the same time. The bar charts show the Spearman’s rank correlation coefficients (rs) for the levels of gene regulation during stress adaptation in relation to the levels of changes in H3K18ac, H4K16ac and Gcn5 (left panel) as well as for levels of up-and down-regulation during the same period in relation to the levels of changes in Gcn5 (right panel). Statistically supported correlation coefficients (*) as well as coefficients lacking statistical support (ns) are indicated. The p-values for comparison of regulation level with H3K18ac, H4K16ac and Gcn5 levels were 2.20E-16, 0.32 and 0.64 and for comparison between the levels of up- and down regulation with Gcn5 levels they were 4.81E-4 and 0.71, respectively. **B** Significant correlation between transcript abundance and levels of H3K18ac, H4K16ac and Gcn5. Annotations are as for part **A**. The p-values for comparison of transcript abundance with H3K18ac, H4K16ac and Gcn5 are 2.20E-16, 1.73E-05 and 2.20E-16, respectively. **C** No significant correlation between gene regulation level during stress adaptation and transcript abundance. Scatter plot showing Log2 transformed fold change values for the stress adaptation period plotted against Log2 transcript abundance values. The Spearman rank correlation coefficient is 0.004 (p=0.77).

Gcn5 has previously been implicated in nucleosome depletion and has the capacity to interact with acetylated histones via its Bromo domain [21]. With this in mind, we considered whether Gcn5 might be specifically associated with histones in the H3 depleted region of cluster 1 genes which show a transient increase in H3K18ac during stress adaptation (Sample B). Figure 6D shows that there is a transient increase in Gcn5 levels in relation to H3 levels in the histone depleted region of cluster 1 genes during stress adaptation (Sample B). Furthermore, Gcn5 and H3K18ac levels are transiently reduced for the down-regulated genes in cluster 2 genes at the same time point. We conclude that transient changes in the average level of histone associated Gcn5 and H3K18ac in promoters reflect transient changes in gene regulation that occur during adaptation to stress. We next determined whether the promoter levels of Gcn5, H3K18ac and H4K16ac were significantly correlated with the regulation of genes that were induced or repressed by at least 1.8 fold during stress adaptation. Figure 7A (left panel) shows that there is a highly significant tendency for the promoter peak H3K18ac level to correlate with the direction and level of gene regulation. However, we were not able to find significant support for correlation between gene regulation level and the levels of Gcn5 or H4K16ac at the same time period. To investigate this more closely, we divided the set of regulated genes into two sets of up- and down-regulated genes, respectively. No significant correlation of either of these sets with H4K16ac was found but a significant correlation of Gcn5 levels with up-regulated genes was found (Figure 7A, right panel). The inability to show a correlation between Gcn5 levels and the level of down-regulation may be due to technical difficulties associated with measuring low levels of Gcn5 enrichment. We conclude that changes in the promoter levels of Gcn5 and H3K18ac represent an important mechanism that regulates the activation of many genes.

H3K18ac has previously been regarded as a histone modification associated with highly expressed genes [22]. This is true for the present study (Figure 7B), where the level and significance of the correlation is similar to that shown for H3K18ac with regulated genes. There is also a significant correlation between transcript abundance and both Gcn5 and H4K16ac levels in the promoter peak. Figure 7C shows that there is no observable correlation between transcript abundance and the level of gene regulation (rs = −0.004, p = 0.77) and thus Gcn5 and H3K18ac appear to function together both to maintain the high expression level of highly expressed genes and to activate transcription of up-regulated genes. The molecular mechanism involved could be the same in each case (see Discussion).

## Discussion

Here we show that stress adaptation is a useful approach for studying the functions of HATs, which often show high levels of functional redundancy, making them difficult to study using mutant analysis in steady-state growth conditions. During stress adaptation when gene regulation activity is highly elevated, two clearly distinct genome-wide functions of the Gcn5 HAT are revealed. First, Gcn5 plays a gene regulatory role at promoters. Transient increases in Gcn5 and H3K18ac are well correlated with transiently reduced histone density in promoters and changes in Gcn5 and H3K18ac levels are also significantly correlated with the levels of gene regulation. Second, Gcn5 plays a transient role on the ORFs of long genes during stress adaptation, which is not generally correlated with gene regulation. This role probably involves the HAT activity of Gcn5, which is required for transcriptional elongation.

H318ac has mainly been regarded as a histone modification associated with highly expressed genes [22]. We show that a peak of H3K18ac in promoters is correlated with a similar peak in Gcn5, suggesting that Gcn5 is at least in part responsible for maintaining the high H3K18ac levels on highly expressed genes. However, highly expressed genes tend to be constitutively expressed and do not overlap significantly with the group of genes that are transiently regulated during stress adaptation. We observed that the co-localisation of Gcn5 and H3K18ac at the promoter region is correlated with the transient gene regulation during stress adaptation. The results suggest that similar mechanisms involving Gcn5 and H3K18ac may be involved in both maintaining constitutive high expression and activation of increased gene expression. The mechanism is likely to involve de-stabilisation of key promoter nucleosomes as has been observed for individual genes previously [21]. Our findings suggest that Gcn5 and H3K18ac levels are involved in gene regulation activity at a genome-wide level. This conclusion is consistent with bioinformatics findings that show high connectivity between H3K18ac and transcription factors [22]. Other highly connected modifications are H3K27ac, H2BK16ac, H3K23ac and H3K9ac. Thus the results we show H3K18 may reflect changes occurring at other acetylation sites that occur simultaneously. Consistent with our findings H4K16ac was very poorly connected to transcription factors [22] perhaps due to the important general role it plays in establishing boundaries between euchromatin and heterochromatin [23,24].

An interesting aspect of our results is that the stress adaptation associated changes in the promoter levels of Gcn5 are only evident if we measure Gcn5 levels in relation to histone levels. This is because the transient increase in Gcn5 and H3K18ac during stress adaption is accompanied by a simultaneous transient reduction in histone levels within the promoter, as measured by H3. This global trend is consistent with mechanistic studies of individual genes where acetylation of H3K18 has been shown to precede nucleosome loss at the transcription start site [21]. It is possible that activators recruit Gcn5 to promoters, as has been demonstrated in vitro [25,26], but that the residence time of Gcn5 on promoters is controlled by interactions with histones, either via active-site interactions with substrates during acetylation or more likely via interactions between the Gcn5 bromodomain and acetylated lysines. Interestingly, the Gcn5 bromodomain interacts with H4K16ac [27], the levels of which are much more mildly affected during stress adaptation than H3K18ac.

Measurement of Gcn5 levels in relation to histone levels may be significant in another context. Several reports have shown that Gcn5 levels (not corrected for histone levels) are elevated on a subset of repressed genes under different stress conditions [7,10,11,28] and a similar tendency is seen for the down-regulated genes in this study. This observation is somewhat unexpected given the documented role of Gcn5 as a transcriptional activator and to our knowledge no mechanism for Gcn5 mediated gene repression has been reported. Interestingly, histone density increases transiently in the class of repressed genes during stress adaptation and when this is taken account there is a clear transient reduction in the amount of Gcn5 in relation to histones within the promoter peak, which correlates well with the transiently reduced levels of H3K18ac observed for the same genes. We conclude that studying the chromatin associated levels of Gcn5 and perhaps other histone-associating proteins in relation to histone levels, as has been widely accepted for covalent modifications of histones [21], may provide a useful complement to existing approaches in functional studies of histone associating proteins.

Our data show that histone density tends to be higher on gene ORFs than on promoters and that H3K18ac and H4K16ac tend to be lower for the longer genes. Nucleosomes cause a considerable obstacle to elongating RNA polymerase [29] and their presence in ORFs has been shown to be important for suppressing aberrant intra-genic transcripts, which might be expected to interfere with the transcription of bona fide gene transcripts [30,31]. The density of histones and their degree of hypoacetylation on ORFs increase as a function of gene length. This might be expected, since the density of elongating RNA Polymerase on ORFs that is required to produce a given number of transcripts should be lower for longer genes. Consequently, the proportion of ORF chromatin that is subject to elongation-related hyperacetylation and nucleosome eviction will be lower for long genes than for short genes. Our results show that the HAT activity of Gcn5 is important specifically under conditions of nucleotide depletion (induced by MPA treatment) that limit transcriptional elongation. This result builds further on previous observations suggesting a role of Gcn5 in transcriptional elongation [12] and is consistent with the role that has been suggested for Gcn5 and other HATs in transiently opposing the inhibitory nature of ORF chromatin during transcriptional elongation [32,33].

The transient role of Gcn5 on long genes is unclear. Interestingly, mechanistic studies using individual artificial genes have shown a specific role of Gcn5 in the efficient transcription of long genes and that RNA Polymerase II density in gcn5Δ mutants is reduced at the 3′ end of long genes but that there is no difference at the 5′ end of the same genes nor in short genes [14,19]. Taken together with our results, this suggests that long genes are more susceptible to pausing and loss of elongating RNA Pol II prior to transcriptional termination and that Gcn5 is particularly well adapted to prevent this. The fact that Gcn5 localisation is not particularly correlated with gene length in normally growing cells suggests that there is significant redundancy between HATs with respect to this function, and that this special Gcn5 role is only revealed under conditions of highly elevated transcriptional reprogramming such as those seen during stress adaptation. In conclusion, by studying cells during stress adaptation we have been able to unveil two independent functions of Gcn5 that are not evident in cells growing under steady- state conditions. This represents an approach to studying redundant protein functions that is complementary to classical approaches using mutations individually or in combination.

## Conclusions

We show that reduced levels of redundancy during stress adaptation provide an opportunity for characterizing the genome-wide roles of redundant protein families in gene transcription. Gcn5 plays a gene regulatory role at many activated gene promoters, which is correlated to increased levels of H3K18ac. Gcn5 plays an unrelated genome-wide role on long gene ORFs, which is not correlated to gene regulation and probably involves acetylation of a target distinct from H3K18. Interpretation of the results shows that the levels of chromatin associated proteins can be considered in relation to histone levels as a valuable complementary approach to classical analysis methods. Analysis of data in this way appears to dispel previous indications that Gcn5 plays a role in transcriptional repression.

## Methods

### Strains, plasmid and growth conditions

The Gcn5-myc tagged strain (By4742, *MATα, his3-1, leu2-0, lys2-0, ura3-0* Gcn5- MYC13-KanMX6) is from [34]. Cells were cultivated at 30°C in YPD medium (1% yeast extract, 2% bacto peptone and 2% glucose) to a log phase density of 1 × 10^7^ cells/ml (sample A collection), then and diluted to 5 × 10^6^ cells/ml and subjected to stress by adding a equal volume of 30°C pre-warmed YPD medium containing 2M KCl. Sample B was collected 1hr after dilution. Growth in KCl- containing medium was continued until cells reached a density of 1 × 10^7^ cells/ml (sample C collection). Cells were pelleted and re-suspended in 30°C pre-warmed YPD without KCl at a density of about 2.5 × 10^6^ cells/ml. Sample D was collected 1hr after the medium change. After 2–3 generations of growth sample E was collected. Cell number was counted every hour. 100ml of Sample A-E with around 5×10^8^ cells were collected, one third of each sample was pelleted and frozen immediately in liquid nitrogen for RNA extraction to be used in gene expression profiling; the other two third of the each sample was pelleted and immediately processed by 1% paraformaldehyde fixation and frozen for ChIP-chip experiment. Two replicate cultures were grown on different occasions.

Details of *S. cerevisiae* strains lacking *GCN5* or expressing Gcn5 derivatives containing substitution mutations in the HAT domain are described elsewhere [7]. The strain lacking the TFIIS transcriptional elongation factor *(BY4742; Mat a; his3D1; leu2D0; lys2D0; ura3D0; YGL043w::kanMX4)* was obtained from Euroscarf [35] Spotting assays were performed by spotting 5-fold serial dilutions of cultures with a minimum cell density of 10^6^/ml. Cells were cultivated on synthetic complete medium (Bacto- yeast nitrogen base 0.67%, glucose 2%, bacto-agar 2%, amino acid mix 0.2%), with or without mycophenolic acid (MPA) at a final concentration of 30 μg/ml, or with MPA plus Guanine (final concentration 100 μg/ml).

### Gene expression profiling and ChIP-on-chip microarray experiments

Affymetrix GeneChip® Yeast Genome 2.0 Arrays were used for expression profiling. RNA was prepared by the hot phenol method as described previously [36]. Probe labeling and hybridization were performed according to the Affymetrix manufacture’s protocol.

Affymetrix GeneChip *S. cerevisiae* Tiling 1.0R arrays were used for ChIP-on-chip experiments. Antibodies directed against H3K18ac (ab1191), H4K16ac (ab61240) and Histone H3 (ab1191) were obtained from Abcam and were used at a 1:100 dilution for Chromatin Immuno-precipitation experiments. The antibody directed against Gcn5-myc was obtained from Sigma (M5546) and used at a 1:50 dilution in ChIP experiments. The ChIP-on-chip procedure was as described previously [12]. Probe labeling and hybridization were performed according to Affymetrix manufacture’s protocol.

### Data analysis

Gene expression profiling data analysis was processed with the RMA method using Affymetrix Expression Console software. Fold changes were obtained by comparing expression level to that under normal growth conditions (Sample A). K-means clustering [37] was used to identify different gene expression groups. Cluster dendrogram of conditions is performed by the core function in R packages (http://www.R-project.org). GO ontology analysis was performed using GOminer software [38] as described previously [7].

Raw ChIP-on-chip tiling array data from Affymetrix tiling arrays (CEL format) were analyzed using Tiling Analysis Software (TAS) from Affymetrix to obtain genome- wide ChIP signal (bar file). The data for the 5 samples (A-E) were normalized to the have the same median value. Average gene analysis was done using a procedure modified from [18]. Briefly, the signals corresponding to the ORF region of each gene in *S. cerevisiae* were divided into 20 equally sized bins. Signals from both the 5′IGR and 3′IGR were divided into 9 equal-sized bins. The 5′IGR was defined as a region starting at the middle of upstream intergenic regions and ending at the nucleotide preceding the initiation codon. The 3′IGR was defined as the region starting immediately after the stop codon and ending at the middle of the intergenic region. The Java code for average gene analysis is available by request. Correlation analysis was performed by calculating the Spearman rank correlation coefficient and associated p-value using the basic function in R package (http://www.R-project.org).

Since the modification of histones can only occur in chromosomal regions in which relevant histones are present, it is common practice to view levels of histone modifications in relation to histone levels, as used in this study [21]. While this approach is useful, it also has limitations since in the extreme case, where no histones are present in a region, the denominator in the normalization would be zero, leading to the interpretation that modification level would be infinitely high. The approach should thus be used with caution and as a complement to classical microarray normalization methods that do not involve correction for histone density, which are also used in this study.

### ChIP-qPCR

Bio-Rad iQ™ SYBR green super mix (Cat. No. 170–8880) was used for ChIP-qPCR reactions, PCR reactions were conducted using a Bio-Rad “i cycler” Thermal cycler with the following settings: 95°C for 3min, then 40 cycles at 95°C for 15 sec, 57°C for 30 sec and 72°C for 30 sec. Internal control genes for which Gcn5 levels were not expected to change were chosen based on the ChIP-chip data. The primer sequences for control genes and long genes are described in Additional file 6.

### Data access

The project data is available at Gene Expression Omnibus (GEO) http://www.ncbi.nlm.nih.gov/projects/geo under accession number: SuperSeriesGSE 36601. Subset series GSE36599 is the expression profiling array data and subset series GSE36600 is the tiling array data.

## Abbreviations

HAT: Histone transferase; HDAC: Histone deacetylase; GEO: Gene Expression Omnibus; GO: Gene Ontology; MPA: Mycophenolic Acid; TAS: Tiling Analysis Software; TSS: Transcription start site

## Competing interests

The authors declare that they have no competing interests.

## Authors’ contribution

YX-F designed the experiment, performed the experiment and performed the data analysis. She interpreted results and contributed to writing the paper. AW conceived the study, interpreted results and contributed to writing of the paper. JH wrote scripts to solve problems with the tiling array data analysis. TB proofread the version to be published. All authors read and approved the final manuscript.

## Supplementary Material

Additional file 1 Shows ChIP-qPCR results for Gcn5 level changes between stress adaptation and normal condition for long genes. Click here for file

Additional file 2 Shows the average acetylation levels of different gene length groups for 11 lysine sites. Click here for file

Additional file 3 **Shows gene length associated differences in average levels of H3K18ac and H4K16ac at ORFs in samples taken during the stress and recovery growth regime with H3 normalisation (in comparison with Figure **2** which is without H3 normalisation).** Click here for file

Additional file 4 Shows enriched Gene Ontology groups for regulated genes during stress adaptation and recovery regime. Click here for file

Additional file 5 Shows the transient changes in gene expression during the stress and recovery growth regime in correlation with the transient changes in average levels of Gcn5 and histone acetylation at promoters. Click here for file

Additional file 6 Shows the primer sequences for the ChIP-qPCR. Click here for file
