# Supplementary material for: Distinct roles of the Gcn5 histone acetyltransferase revealed during transient stress-induced reprogramming of the genome
Source: BMC Genomics. 2013 Jul 16;14:479. doi: 10.1186/1471-2164-14-479 (PMC3723427; doi:10.1186/1471-2164-14-479)
Supplement: Additional file 1 — Shows ChIP-qPCR results for Gcn5 level changes between stress adaptation and normal condition for long genes. [file 1471-2164-14-479-S1.pdf]

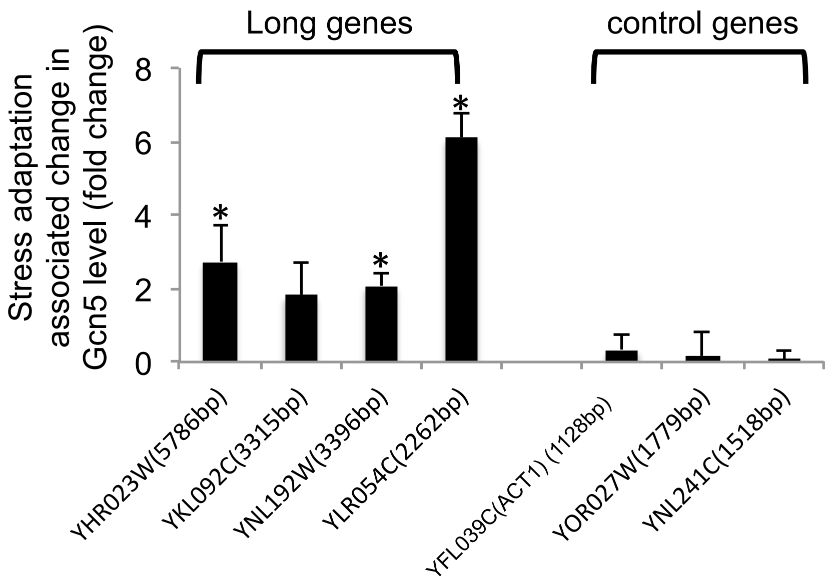

Long genes have higher Gcn5 level under stress adaptation by ChIP-qPCR. **F**old change of Gcn5 level between stress adaptation conditions (sample B) and normal conditions (sample A) are plotted. Gene length for long genes (left panel) and controls (right panel) are indicated. * $p<0.05$, t-test of fold change significance for long genes compared to the *ACT1* gene (YFL039C)
