# Supplementary material for: Distinct roles of the Gcn5 histone acetyltransferase revealed during transient stress-induced reprogramming of the genome
Source: BMC Genomics. 2013 Jul 16;14:479. doi: 10.1186/1471-2164-14-479 (PMC3723427; doi:10.1186/1471-2164-14-479)
Supplement: Additional file 2 — Shows the average acetylation levels of different gene length groups for 11 lysine sites. [file 1471-2164-14-479-S2.pdf]

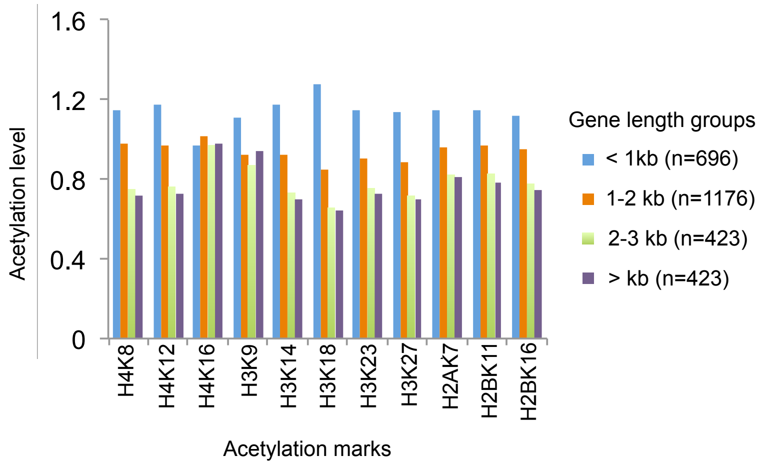

Average acetylation level at ORF is negatively correlated with gene length for most lysine sites. Average acetylation level at ORF is plotted for different length groups as indicated (arbitrary unit) . The number of genes in each group is shown in parenthesis. Data are from (Pokholok et al., 2005)
