# Supplementary material for: Distinct roles of the Gcn5 histone acetyltransferase revealed during transient stress-induced reprogramming of the genome
Source: BMC Genomics. 2013 Jul 16;14:479. doi: 10.1186/1471-2164-14-479 (PMC3723427; doi:10.1186/1471-2164-14-479)
Supplement: Additional file 3 — Shows gene length associated differences in average levels of H3K18ac and H4K16ac at ORFs in samples taken during the stress and recovery growth regime with H3 normalisation (in comparison with Figure 2 which is without H3 normalisation). [file 1471-2164-14-479-S3.pdf]

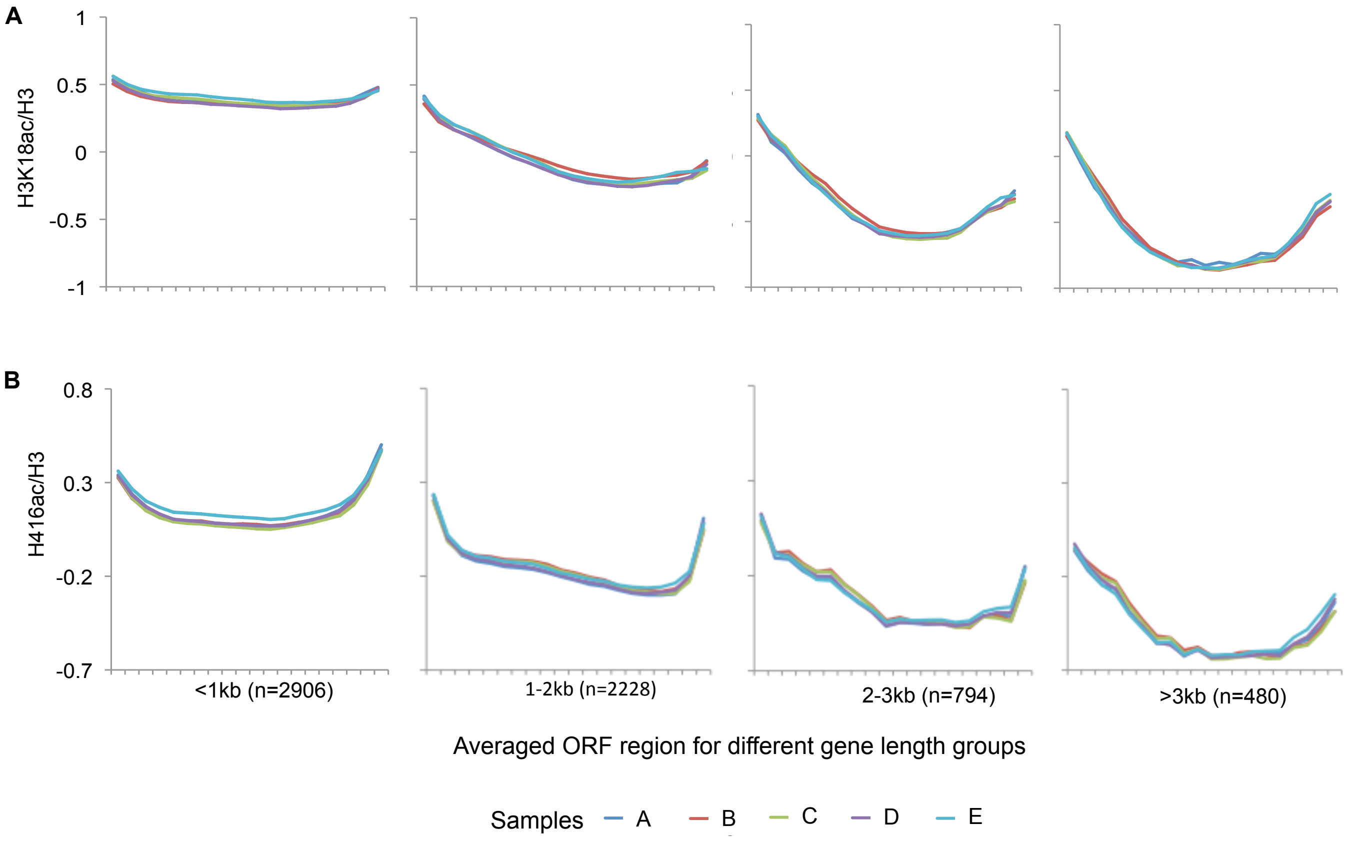

Genome-wide gene length associated differences in average levels of Gcn5, H3K18ac, H4K16ac and H3 at ORF in samples taken during the stress and recovery growth regime. The relative average levels (arbitrary units) of H3K18ac (A) and H4K16ac (B) relative to histone H3 at ORF are plotted. The arbitrary units are independently defined for ChIP antibodies. The criteria defining the gene-length groups are shown. The number of genes in each group is shown in parenthesis. Line colors represent different samples taken during the stress and recovery growth regime.
