# Supplementary material for: Distinct roles of the Gcn5 histone acetyltransferase revealed during transient stress-induced reprogramming of the genome
Source: BMC Genomics. 2013 Jul 16;14:479. doi: 10.1186/1471-2164-14-479 (PMC3723427; doi:10.1186/1471-2164-14-479)
Supplement: Additional file 5 — Shows the transient changes in gene expression during the stress and recovery growth regime in correlation with the transient changes in average levels of Gcn5 and histone acetylation at promoters. [file 1471-2164-14-479-S5.pdf]

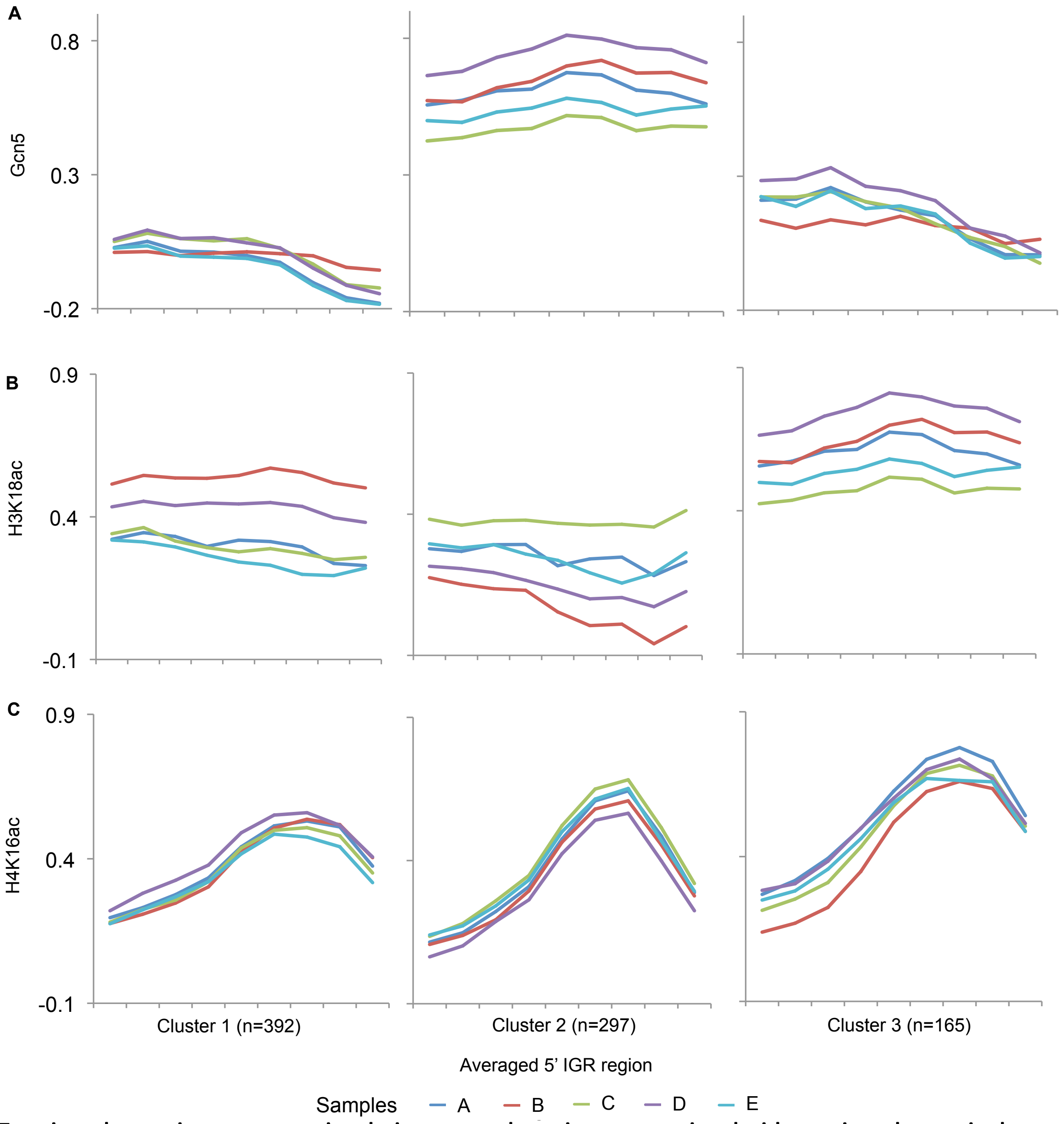

Transient changes in gene expression during stress adaptation are associated with transient changes in the average levels of histone H3K18 acetylation at promoter. The average levels (arbitrary units) of H3K18ac (A), H4K16ac (B), and Gcn5 (C) at promoter are plotted. The arbitrary units are independently defined for each ChIP antibodies. Clusters 1-3 are the same as those identified in Fig. 4 and the number of genes per cluster is shown in parenthesis. Line colors represent different samples taken during the stress and recovery growth regime.
